# Interlude 1: Crablike Collective Moves

**DOI:** 10.1080/09528822.2024.2339144

**Published:** 2024-05-13

**Authors:** Rex Butler, Khadija von Zinnenburg Carroll, Renate Dohmen, Stacey Kennedy, Astrid Korporaal, Marie Meyerding, Barbara Preisig, Azadeh Sarjoughian, Pfunzo Sidogi, Deniz Sözen

**Keywords:** Azadeh Sarjoughian, Khadija von Zinnenburg Carroll, Barbara Preisig, Deniz Sözen, Rex Butler, Renate Dohmen, Astrid Korporaal, Pfunzo Sidogi, Marie Meyerding, Stacey Kennedy, archive, polyphony

## Abstract

This interlude follows the AAH conference, when all of the authors who contribute to the special journal edition are present. Their wide-ranging conversation covers the idea and possibilities of a polyphonic history of art. This interlude is a starting point, a jumping off point from where a group of authors, researchers and artists reflect on new ways of thinking about, and new ways of presenting, art history. Their conversation develops an ambition to move away from the well-trodden and often reductive methodological frameworks of art history, as they debate ideas around transnationalism, globalism, artist-centric art histories and the idea of artist as author. This interlude is about collective endeavours, both by the subjects being discussed in each paper, and by the authors who are writing about them.


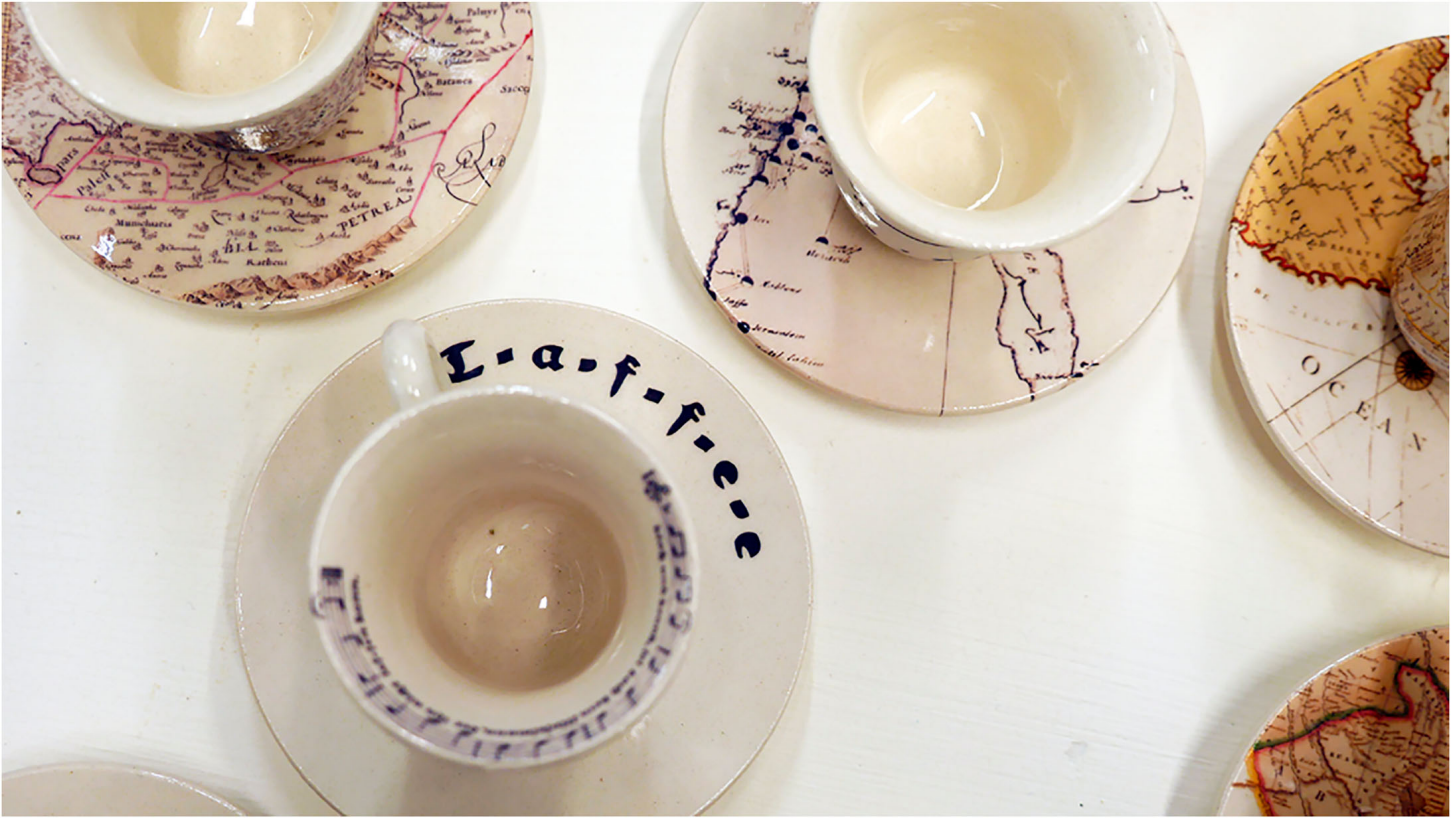


**Deniz Sözen, *Kahvehane*, 2016, (detail), set of nine (‘Turkish’) coffee cups and saucers, transparent glaze and transfer print on Kütahya ceramics, edition of 7 + 2 AP, produced at the Ceramics Research Centre, University of Westminster, London**

**Khadija**
Is polyphony a methodology for you, Barbara? How did you come to it and where is it going?

**Barbara**
I am using the concept of polyphony as a figure of speculation. It urges me to stretch my methodological framework. How might polyphonic writing in art history sound? How freely can I play with (historical) sources to make audible the polyphonic reception of an exhibition? How to situate the author – myself – within the polyphony? It’s trial and error, and I would be very interested in having many such experiments on different levels of polyphony in this special issue and then maybe afterwards trying to find a methodological approach from these experiments, and not the other way around.

**Rex**
We actually do have something in common across approaches that might seem different. You know for instance Barbara is writing experimentally, and ours is more historical, but the thing that our papers have in common and what pushes us to hang on to the concept of polyphony, is that we all think about refusing universalism/globalism with its one voice speaking from nowhere about everybody. Instead, what we are doing is a translocal connecting of two things – two voices at once, two places at once, no matter which one is in the first place and which one in the second. It is about two places speaking to each other as in a song, and it’s very different from the usual globalist art history. What we are doing is avoiding the theorisation of everything from nowhere and I think that’s a strong aspect of our papers.

**Khadija**
It’s interesting that ‘transnationalism’ has been appropriated as a theme through which to theorise, cover and assimilate all non-Western art.

**Rex**
I think a methodological question, a bigger issue, would be the one we’ve touched on, which is when the idea of a central Western canon dissipates, how do people continue to speak to each other when we all have are our own individual stories? If we’re all writing from where we are particularly and we don’t have this one thing that we all have to automatically defer to, how do we remain interested in each other? What is the relevance of another person’s story to us?

**Khadija**
This reminds me of a discussion around minor universality or negative universality that French philosophers and German anthropologists are having at present, to find commons that do not assimilate minorities into universals, as another way of discussing what Elizabeth Povinelli calls counter-reformation in our article in this special issue.^1^

**Barbara**
This also brings me to the question of oral history. An interview can be very polyphonic… or not. It depends on how a question is being posed and on how open we are to receiving ambiguous responses that may multiply or confuse the question instead of answering it. Are we looking for one voice are we looking for many voices to speak for today? And so on.^2^

**Khadija**
I’m excited about your theatrical staging because I was instinctively experimenting with something similar, albeit half-baked, in graduate school. I remember presenting a dialogue between Max Ernst and Denis Diderot for my first Methods course presentation where everyone at Harvard was trying to prove how erudite they were. People were laughing because my Mexican colleague Ana Pulido Rull was Diderot in a fittingly heavy accent, and I was Ernst. I wrote a script we both read, about a work of art by Ernst. The teacher, Ewa Layer Burkhart, said, ‘That’s good, but next time can you do it [which meant the art history, the analysis, in other words] just to show you can.’ At the time I stopped wasting my energy; the year before, I had to battle to get a similar writing experiment published by the journal *Word & Image*.^3^ They thought having Winckelmann and a cast of characters including speaking artworks like *Laocoön* on stage was a weird way to start an academic article. I would hope, by now in the history of art, we have shown what we can and can’t do. This special issue takes that for granted, so we can move on with new experiments, networks, tables and tones, be they round, octagon or otherwise. As Deniz says, we have so many positions often within us already, how do we articulate those and build a space for them here on the page?

**Pfunzo**
As art historians, when we engage with untold narratives, I think it’s a continual process of invention. This applies to how we, as art historians or co-writers, are always engaged in inventing histories, stories, whatever it is we’re working on. I really like that idea. I wonder if that could be one of the threads that runs through all our writings – this concept of inventing the stories.

## Positionality of Author-reader and Artist-centric History

**Rex**
There’s a paper in the art historiography of Australia written by Ian Burn, a very well-known conceptual artist, a famous polemic for an artist-centric art history, and maybe it sounds weird but that could be one of the things that everyone’s paper has in common.^4^ An emphasis on our history told by artists. Art historians often exclude this sort of scene- or art-making and that seems to be something that lots of people here emphasise. The question is, what would an artist-centric art history be like? That’s one way of summarising the arguments of the various papers. One of the things that several of our papers have in common is the postulation of an artist-centric history, which would be more about the life experiences of certain groups of artists rather than retrospective justification of what has largely already entered an art museum. Ian Burn’s essay is a very early example of something that lots of people are now writing about, which is the idea of reversing the poles of our art history and seeing it from the point of view of the practitioners at the beginning rather than the art historians at the other end.

**Figure:**
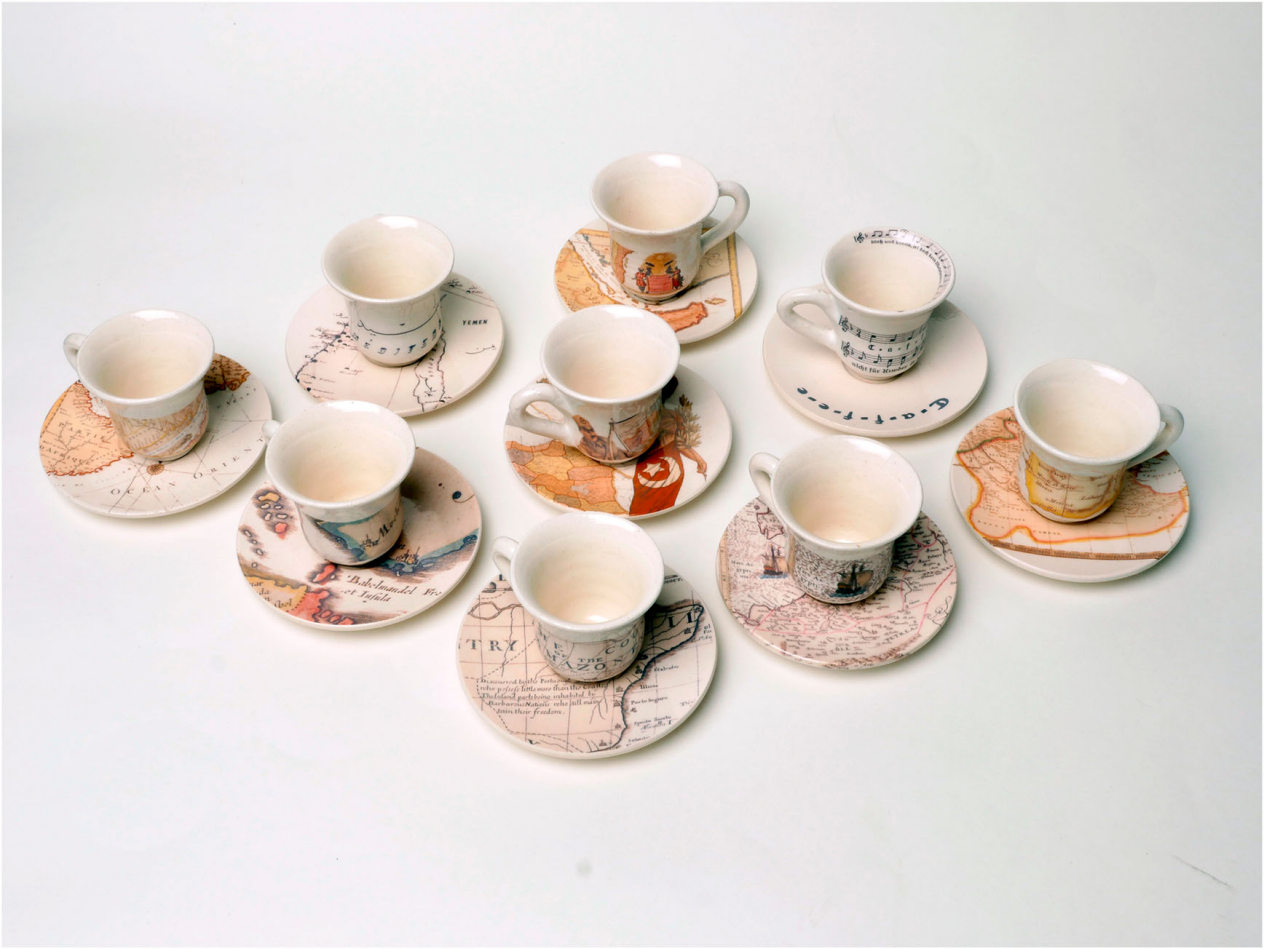
Deniz Sözen, *Kahvehane*, 2016, set of nine (‘Turkish’) coffee cups and saucers, transparent glaze and transfer print on Kütahya ceramics, edition of 7 + 2 AP, produced at the Ceramics Research Centre, University of Westminster, London

**Khadija**
Because artists are always seen as kinds of geniuses that can’t read or think, and, on the other hand, the art historian traditionally must not act like an artist and write in a way that might be associative or non-linear, fictitious or experimental. I think that’s what we’re also challenging from other angles. Pfunzo’s is an important paper that resonates with other regions, like India or Australia, where artists (making prints or otherwise) did not relate to their authorship with the conception of the self as artist that we now assume and also project back on to these printmakers.

Another example are the Indian Viswakarma or Asari sign painters who work on cinema billboards, with whom I made an exhibition for Platform Gallery (Melbourne) in 2003. Anonymous artisans, they operated as family guilds and did not conceive of themselves as individual artists, although they were also hierarchical, with the father usually running the workshop. An abbreviated polyphony of the images we produced shows how different they were in style and voice.

**Figure:**
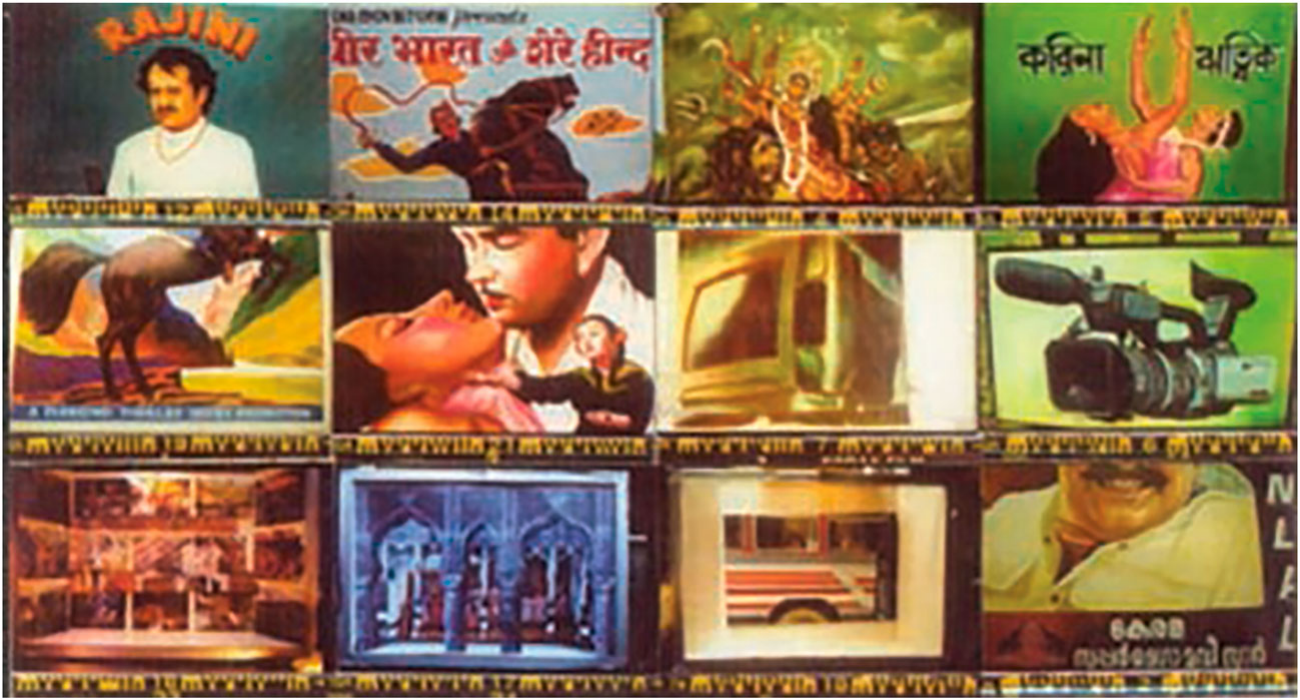
Khadija von Zinnenburg Carroll, Clemens Fuertler and sign painters, invitation card featuring twelve of the billboards hand-painted in various workshops around India, Platform Gallery, 2003

Later, while at the University of Cambridge’s Museum of Archaeology and Anthropology, I worked on the collection and exhibition entitled ‘The Power of Paper: 50 Years of Printmaking from Australia, Canada and South Africa’, which traced how in former British dominions colonised peoples and Indigenous communities began to represent themselves through art in modern media. Since the 1960s Indigenous and Black artists from the Arctic to the Australian desert, the country to the city, have depicted their culture, history and struggle through prints. We foregrounded visions of place, custom and history in settings that are at once profoundly different yet linked by empire and the politics of decolonisation.^5^

**Figure:**
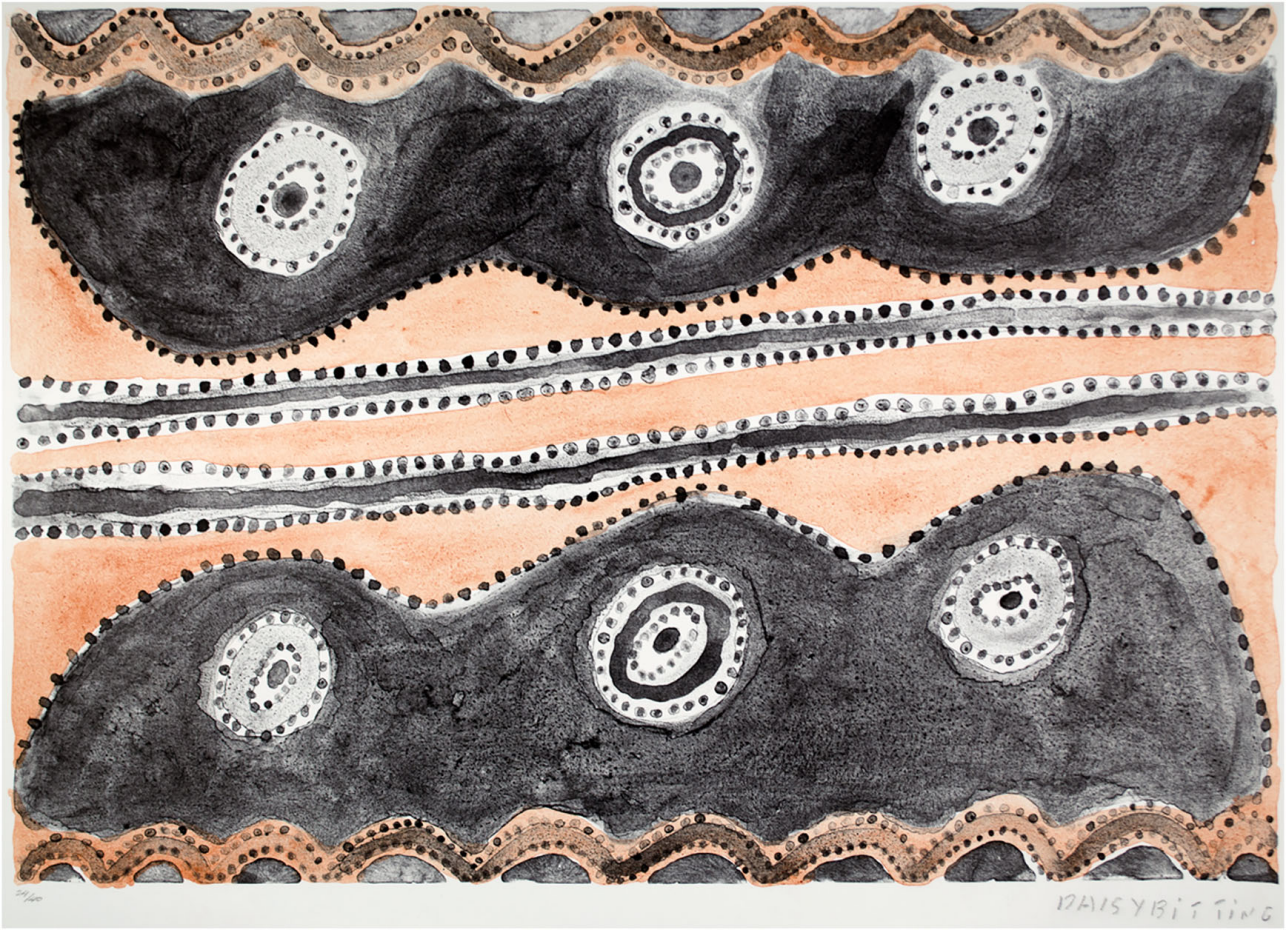
Daisy Bitting, *Wirrpahloo*, 2001, lithograph, Museum of Archaeology and Anthropology, University of Cambridge collection and exhibition ‘The Power of Paper: 50 Years of Printmaking in Australia Canada and South Africa’, 2015

**Figure:**
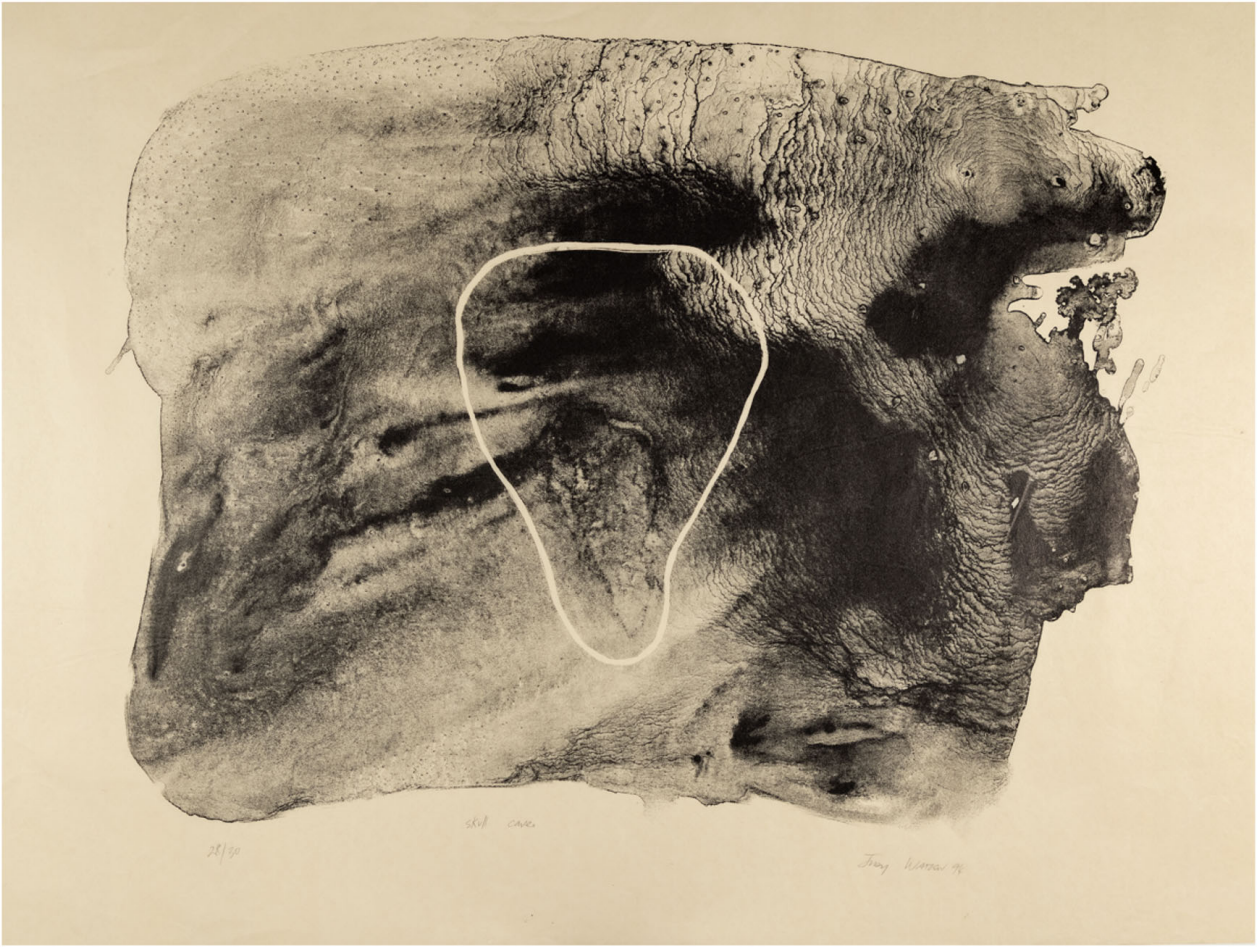
Judy Watson, *Skull Cave*, 1994, lithograph, Museum of Archaeology and Anthropology, University of Cambridge collection and exhibition ‘The Power of Paper: 50 Years of Printmaking in Australia Canada and South Africa’, 2015

**Pfunzo**
The emphasis on an artist-centric art history is fantastic. In my research the artist is important, but more so in how they surface through the artwork itself. Especially in the context of early twentieth-century colonial South Africa, the only evidence of Black artists using the medium of printmaking is images of the artworks that survive in the archive. To go back to Khadija’s provocation around the notion of the artist and authorship, as we understand it in a contemporary sense or Western conception of an artist, it is not always tenable to impose this category on colonial-era artistic histories. Of course, you can start reading into the artworks the ‘imprints’ of some kind of virtuoso artistic style, which speaks to the individual who was imposing their unique aesthetic will on the image. To this end, the artworks reveal a little bit of the artist’s energy. But we cannot find a biography and social history of an individual artist in the same way we tend to find it in classical European art history. All we have are hints of a profile of the artists who might have created these works.

In essence, we have a collective imagination of the individual artist expressing a self-image consistent with the aspirations and influences of the day. But the self-image we see in the art is not so much the artist thinking about their individual self, but the artist channelling the stimulants related to the spaces and communities in which they existed on to their images in some way. The printmaking modality embodied what it meant to exist as a Western-educated Black person – or Black man, since it was male subjectivities that were mostly captured – during that period in South Africa’s history. Printmaking, through the newspaper presses, was one of the very new forms of creative expression that were available to Black artists during the early 1900s. Another point to consider is the very platform where these artworks are archived. Because the images only survive in newspapers, and Black newspapers to boot, they are not considered art. Newspapers are generally not recognised as centres of artistic expression. That said, there is a need for revisionist explorations that intentionally look away from the museum and gallery spaces and focus on the popular press, to find forgotten creative narratives of both the artists and their artworks.

**Deniz**
I wanted to add that ‘polyphonic’ also has ‘phonic’, as in ‘sound’, in it, indicating the oral and the voice, and indeed the artist’s voice is often silenced in the history of art. And then, of course, there is this whole debate of who is allowed to speak (for whom) – and the question, can the subaltern speak? – and whose voices are heard. I think all these debates also filter in.

**Marie**
That is the first question people ask when I tell them about my research. They ask, why is it on Africa? And I think it’s interesting to reflect on that. What this question implies is a limitation of what I can write about, and this in turn raises questions for me about the translatability of experience. So, for example in the German context, can I write about GDR artists or can I only write about West German artists? And how do I reflect on this? I think it is very important to reflect on the historical structures of our discipline, paying attention to language and to readership through thorough contextualisation. Through this, I interpret Donna Haraway’s situated knowledges to reflect on my own positionality. When thinking about my interviews with artists, for instance, I ask myself, how can I translate these interviews into text? How would that be possible when so much depends on personal relationships, on the visual, on things unsaid, on trust? I think if I wanted to write out an interview with an artist such as Mavis Mtandeki, for example, there would be a lot of difficulties in putting it into text.

**Khadija**
Why difficult?

**Marie**
First of all, we repeat a lot in our conversations in order to understand each other, because English is neither my nor her mother tongue. Also, over time I have acquired a lot of knowledge about the things Mavis Mtandeki refers to that would not be obvious to anyone else. For instance, I organised a discussion between students of the University of Zurich and Mtandeki as a photographer, and while she was speaking I realised that a lot of the things that she referenced would make no sense to the students because it is very localised knowledge about different struggles that took place in the townships around Cape Town. And then I wondered what the students took away from that conversation, because they probably had a very different perception of that conversation than I did.

**Khadija**
I think about the way Rex has done this in his paper and I understand the problem. Maybe I’m biased because I’m interested in Australian art history, but at first Rex’s paper does seem extremely localised, with loads of names and very specific references. Yet it’s the art of how you write that local history, how you keep that going as a narrative that draws in a reader who doesn’t care about a national art history or Australia or anything to do with that, but thinks ‘How strange that there were these groups of people in London, they remind me of my people.’ I mean, that there’s something common or archetypal about the situation, not just for Australians. And yet you have specific actors playing out those roles. Obviously, if you do it like Barbara, you create specific positions and they are literally the same anywhere, it doesn’t have to be in Bern, it could be London or Melbourne or anywhere – I mean, no one complains about the Swiss-German there, but then no one would complain here that the gallery discourse is in a local Australian dialect that no one outside understands. So much of that provincialism is similar around the world.

**Rex**
And that would be nice because it proves that we could end up speaking to each other across our particularities, right?

**Azadeh**
I wanted to say something about Rex’s paper and my experience of reading it, and it just occurred to me when Marie mentioned the difference between those listeners/readers who are aware of local knowledge and histories and those who are not. Rex’s paper is full of names and local references which I didn’t know anything about. At first, I had the concern of not being able to grasp the argument due to my lack of knowledge about Australian art. But then I engaged with the whole narrative, which pushes me to reflect on a particular event, a colony of Australian artists in London, at the same it provokes me to think about a bigger picture that is the cross-border mobility of artists. Sometimes, during my reading, I was encouraged to search for more information about the artists, to get to know them a bit, before returning to the text. Of course, my understanding of the text is not similar or even comparable to that of a reader who knows that context, but it was a joyful practice for me as at all moments of the reading I was aware of the question of context and my own association to this particular one.

**Rex**
Let’s say there’s an uncanny thing happening in art history. My colleague and I have done some research into the Australians who studied at French art schools early in the twentieth century and now Japanese art historians are writing about the history of the Japanese artists who went to French art schools, then went back to Japan and introduced French art to Japan. I mean it’s a powerful story happening all around the world of this kind of trans-globalism happening.

**Khadija**
I was just thinking that there’s a real similarity between what Rex has said and what Azadeh’s research is about. She is looking at the way Iranian artists and their works have been presented in London. This is what happens to expatriate Iranian artists and their experiences, the way that they’re represented: there’s a good connection and something happens; there’s a spark between two people or in the conversation.

**Rex**
Yeah, the story of our writing about the artists in London, it’s just got so many parallels with people from other cultures going to London to study. Indeed, there’s a kind of conversation among artists – they all know of examples of artists from their country going off to study.

**Khadija**
Maybe it’s also a history, then, about migration and why artists migrate. About the incentives for migration, which are not always obvious: London was the centre for the British dominions, plus language and the visa waiver made it easier for Australians to go to London than to Paris. So there are some interesting surprises in the instigation to move, which in a sense, you know, is a kind of crablike, sideways manoeuvre.

**Deniz**
We have previously considered the idea of the nomadic, which resonates with Glissant’s concept of errantry as not necessarily physically going anywhere but also mentally travelling, and ‘the thought of that which relates’.^6^ Also, some of our thinking and thoughts do relate in our papers and that could be something to consider in how we approach the nomadic. And we should also consider the nomadic in relation to the histories of diaspora art. Not necessarily as a sort of neoliberal free-floating we-can-go-everywhere approach, but a look at how it could be a critical practice.

**Rex**
Commonality, connection… 

**Khadija**
That’s why we attend conferences such as AAH (Association for Art History). What’s nice about them is that we find new ideas through each other, which reminds me of anthropologist Michael Herzfeld (‘The Politics of Gesture’), who used to speak at Harvard about gossip and about how gossip is derided as a lowly form of information, but actually when you’re doing fieldwork gossip is essential because that’s where all the really interesting information about relationships comes from.^7^ I was thinking about Marie’s question, about language and repetition, when transcribing interviews from a second language. We might listen and think of how we can edit that kind of material, but often in the repetition or mistake the slippage reveals something.

**Azadeh**
The theme of ‘artist-centric’ or ‘art scene-centric’ led me to think about the art scene in which Shirin Neshat produces her work. As Rex mentioned, writing artist-centric history is more about engaging with the life experiences of artists and their networks; it is an attempt to look at the history of art through artists’ eyes rather than those of art historians or curators. So, thinking about this approach in relation to Neshat, she produces many of her videos in collaboration with other Iranian-American artists, such as Shoja Azari and Ghasem Ebrahimian. I found it interesting when in some of her talks or interviews Neshat refers to herself using the term ‘we’ rather than ‘I’. I’m thinking about whether some of her works can be seen as collective practices, or at least how we can see them in relation to the art scene Neshat lives in. I don’t want to move away from her established position in the artworld as an individual artist, but by considering her continuous collaborations with particular artists, she can be also considered a ‘spokesperson’ for an ‘artist colony’. Of course, this can be seen as a common practice of directors, but what I want to say is similar to what Rex depicted in his paper. We can probably imagine this for communities of Iranian artists in the US, to think about how they live and work together. This is a polyphonic approach. It offers more than a reflection on the sociopolitical context of an artist and her artwork; it is also about the relationships between individual artists and writers.

**Renate**
Bringing out the resonances as papers speak to each other is going to be exciting, and it adds something of a relationality of sorts, developed between issues that may be related, and unfolds uniquely in different geographies reflecting divergent histories, for example of colonialism. Our roundtable meetings have also started a process of resonance building, as while we are having these engagements something is beginning to happen, and the second versions of papers reflect that they have moved in response to our previous conversations. We’re beginning to create something you could call polyphony, and our group discussions are like a large container that acts like a resonance body with papers like a different musical event or notes that are being struck. It is going to be exciting to figure out how to build the music, if you like.

**Astrid**
I was also thinking about the resonance between papers, and particularly between Azadeh’s and Barbara’s papers, where different voices are being performed and brought in. There is something about being labelled a spokesperson, as you describe Shirin Neshat, which has a sense of taking their individuality away. But you point to the way she plays with this role, by making this spokesperson character of herself a site for dialogue and dreaming with multiple voices. This resonates with the feeling I had when reading Barbara’s text as well.

**Azadeh**
Yes, but it’s important to note that attributing the label of spokesperson in this context is often an institutionalised decision rather than an artist’s own choice. However, *Land of Dreams* can indeed be seen as a response to this discourse. What Neshat depicts in this work is also a polyphonic depiction of different stories while relying on the idea of being between two worlds to address and question established dichotomies. The installation sits between fiction and oral history and it’s like a result of dialogical practices within internal and external worlds; for me it recalls Bakhtin’s metaphor of polyphony.

**Renate**
Astrid and I thought it was a trickster move because it is playing with the binaries and not doing the expected. Subverting expectations by switching perspectives in a playful way that speaks volumes, flipping associations of the Orient, North America is a trickster move, a doing that does the unexpected, and does not fall in with the tropes and traditional representation. So that might be another resonance or a connection where one could pull out something as a theme for a discussion (see Interlude 2) between a couple of papers and explore whether what one might call trickster moves is a strategy artists use right now, whatever their background, even if they are not of Indigenous descent.

**Astrid**
And connected to that theme, the subject of the carnival and the reversal or switching of roles, which is also a historical part of western European culture.

**Khadija**
Ah there are so many tricksters amongst us! Think of Barbara’s voices, in her mode of writing history, I think that could be read as another trickster.

## Polyphony in the Archive

**Khadija**
We return to the subject of the archive, because neither Barbara nor I felt like we wanted anything of what was there in the transcript (from our last roundtable).

**Astrid**
Yes, I have been trying to think more about the archive as well, going back to Derrida. I’m thinking about the image and the problematic colonial connection with the image as extracting and capturing something. There is also in this an idea of the archive as capturing and fixing the moment of origin as a kind of proof, as Derrida also talks about the archive in relation to legal matters. I was thinking, how do artists respond to that using images in different ways? And then what this living archive could be, when it’s not about going back to this original moment of proof, or authentic identity, which I think comes back in a lot of the papers. That we’re resisting this kind of authentic identity while still being interested in connections between place and identity or place and cultural expression.

**Renate**
For Kent Monkman, the way notions of authenticity with regard to First Nations peoples have historically been constructed is also a central issue, that is, the circulation of a false identity that becomes a deeply entrenched, popular trope that fixes Indigenous people in the past. It is so well entrenched that is has become a point of identity for Indigenous people, as Gerald Vizenor points out, even though it’s a fabricated and colonial representation. The issue is complex and potent.

**Azadeh**
I was also thinking about polyphonic writing in relation to notions of the living archive and ongoing storytelling. For example, Deniz explores this through her personal journey as a diasporic artist and researcher performing a ritual with multiple references to belonging and calls it ‘the living archive of diaspora’. Through reading her article, I’ve imagined how a polyphonic writing that is supposed to capture or create various voices can be initiated through one body, a body that, for example, is able to perform a meaningful multilingual conversation. Astrid’s paper provides insightful perspective on a collective approach to a living archive, where the shared experiences and stories of a group engage with an evolving, communal history. I think both papers collectively highlight how living archives, whether manifest individually, like Deniz’s experience, or collectively, as in the Karrabing Collective, can be foundational to polyphonic writing. Another relation I see between these two papers is that both demonstrate how diverse voices and stories can be expressed through writing or speaking about moving images.

**Renate**
I really enjoyed the way Deniz situates herself in her paper. It fits in well with what we’re trying to do here, and it also presents the personal dimension, adding something unique. Many of us are kind of engaging from the ‘outside’ so to speak and reflect on the work of an artist. But Deniz, you are both the artist and the author who writes and moves between sides.

**Astrid**
Yes, I agree, and something I was thinking about a lot when reading Deniz’s paper was the relationship between resonance, echoes and embodiment. Maybe it connects to the way I approach peripheral vision as a biological ability, which is not proposed as a universal experience but as a common metaphor to think with. Resonance triggers certain feelings and responses in our bodies, which are not identical but can communicate.^8^ The link between ritual and root that you bring up in relation to the body as an archive is also interesting. It makes me think of a place where things are stored and remembered but that come up in different ways. It is a capacity we all have, but the resonances are different.

**Deniz**
In your text, Astrid, there is also some resonance with the notion of opacity and the mermaid that some can see and others cannot. It can sometimes be a visual thing, but it can also be linguistic opacity. Also, the idea that the story exists in a place at different times and can be perceived from various perspectives contrasts sharply with the modern archival notion where an image of a place anchors it to a specific event and a defined set of relations. I’m very curious about the relation between images, storytelling and English, and how that might be understood differently by First Nations people as opposed to the modern colonial archive.

**Astrid**
To me, this relates to the history of photography and the idea of the image as a document or proof, which registers something as having happened in a particular place and time. But that is only one way to use an image. Inspired by the Karrabing Collective, I’m interested in the image that is part of movement and has an opacity of meaning because it connects different perspectives in movement. The members of Karrabing Collective refer to going to a place and finding a story there, but it is not just one story with a single approach that they find. Everyone has a different version of the story because they approach it in different ways, and it also changes with different experiences and interpretations. I’m interested in the ways the image becomes part of their process of travelling, visiting and remembering, which we could call a living archive, because it keeps the story animated.

**Renate**
This resonates with the issue of orality and storytelling that is raised in my text, and what happens when a story is written down and thus arrested in its flow and movement between the storyteller and respective audiences and fixed and pinned down as a text, which touches back on our discussion of the archive and indigeneity. Oral storytelling always happens in the moment and it’s never the same, but then it is fixed and becomes a representation and part of the archive. Our discussion today raises several questions for me. There is the issue of geographies and how we talk about them and relate them to roots and root identities. Then there is also the question of the native informant and the expert in relation to how to relate to material that does not reflect one’s cultural background: would this be a stance of the quasi-anthropologist as outside ‘expert’? Or, if one speaks from one’s own cultural background, what are the assumptions in terms of being a spokesperson and carrying this burden or a native informant perhaps? What are these relationships? And how do they need to be reconceived or renegotiated? How are they constituted in terms of speaking with or nearby? How are you, Astrid, for example, talking about the Karrabing Collective, from your understanding and point of situatedness. This is why I thought it was good that you started from a personal experience, and was curious about how you then shifted to more of a stance of the expert or transitioned into a more familiar academic or scholarly mode. For you, Deniz, this is different because you’re writing about yourself, so you can move between these positions and play with them, which you do so well. So how do we negotiate these relationships?

**Astrid**
In my dissertation I reflect on the ways other curators and I can learn from artistic practices of collaboration, especially between Western and non-Western, or settler and Indigenous cultural contexts. I’m asking what we can learn from these practices that are questioning colonial constructs of seeing and showing, and what kind of responsibility we have as viewers. Not trying to appropriate Indigenous knowledge or speak for others, but questioning both where colonial modes of thinking need to be resisted in our personal contexts as well as our implication in the continuation of coloniality.

**Azadeh**
Engaging with the notion of geography and location as a kind of situated knowledge, I’m wondering how the Karrabing Collective choose their locations? Are they always going back to a particular place?

**Astrid**
From my understanding, the way Karrabing Collective members speak about it is that both their ancestors, and the spirits or Dreamings of the land, are connected to specific places and geographic formations. When they haven’t been to a certain place for a while, the ancestors and spirits might get upset with them, because they haven’t kept up the relationship. From the point of view of a modern, Western tradition of thought, I think we see our ancestors as located in a radically other space, an inaccessible time zone. But the impression I get from the Karrabing’s films is that their ancestors are still there in these places, at the same time, just in a different plane of reality or visibility. But it’s even more complex since a spirit or ancestor can also move away from a place they have ties to, because they haven’t been visited or because their people have been forced to move. So people might find that they return to a place and the spirits are not there anymore, and they might find that they turn up somewhere else. This also has a bearing on the way they have had to deal with the fact of their displacement within the country. But I think the key thing is that they don’t have to go to the past to speak with their ancestors; or, more accurately, the past is not a separate space. And I think this is not so different from many other cultures, where there is a belief in the connection between place and the memories related to it.

**Azadeh**
Yes, this temporal dimension of the land is fascinating. In the case of Neshat, she often adopts a stereotypical image of an Iranian or Middle Eastern landscape, and the desert is a prominent motif in her work – although the desert landscape can be located anywhere, whether in the Middle East or the US. But the interesting point for me is when one landscape is chosen to refer to another one and therefore a complex interaction between two places and their histories can occur. It’s like expanding the history and connotations of a specific location by engaging with the other one.

**Figure:**
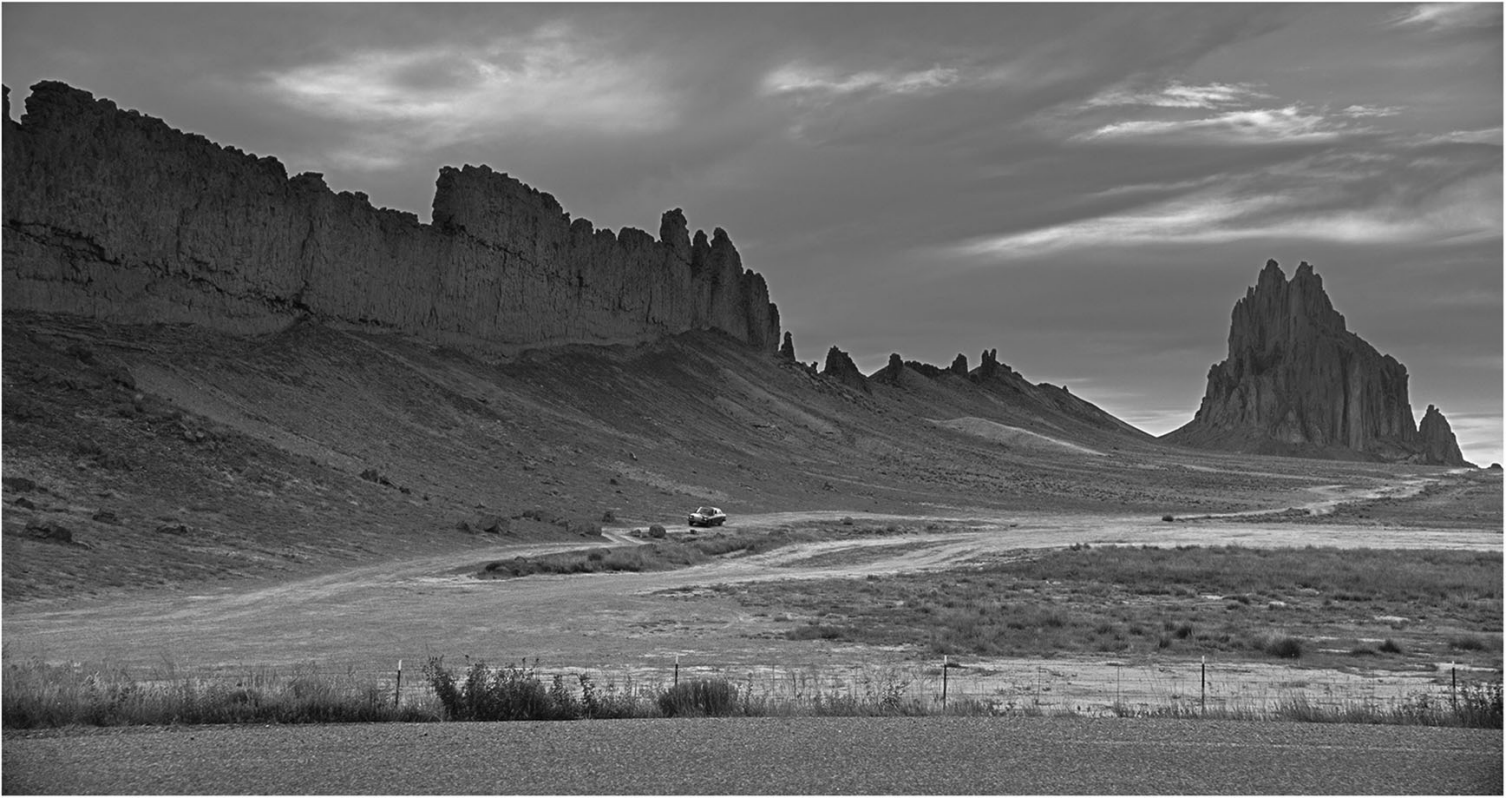
Shirin Neshat, still from *Land of Dreams*, 2019, HD video monochrome, 25:16, © the artist and Goodman Gallery, London

**Astrid**
Yes, that’s very interesting. Although these practices are different, I think they both resist the idea of the universal extractive archive, where things are deposited either physically or digitally and are accessible with the right permissions. There is an attention to the embodied effects of movement and memory that connects specific places, people and memories.

**Barbara**
I think it is important to distinguish between polyphony as an object of research (thinking about what?) and as a method (thinking how, writing how, acting how, researching how?). And I keep asking myself, what is polyphonic art history? I don’t think we should necessarily define the term, but I would like to be more precise on the question: what’s our project here? What are we hoping to achieve, polyphonically, and how? Is the claim for decentralisation of this Euro-American focus already a sufficient condition for polyphony?

**Rex**
Building on that, one of the strengths of our last conversation was the distance we were trying to take on the idea of this kind of ecumenical world art history that tries to theorise everywhere at once from some sort of non-declared perspective; you know, all those histories of ‘contemporary art’ which present a chapter on every country in the world but implicitly are always written from one place. One of the good things about our thinking around polyphony is that, instead of that bland ecumenical balance, we try a different model with transnational connections from one place to another, which is what polyphony is, or can be. That is, what many of the papers have in common is a connection between two places. Someone’s writing from Germany about Africa, or in our case about England from Australia. It’s not any kind of universal globalist history, but actually always a specific set of connections.

**Barbara**
I couldn’t agree more with you, Rex, but can we be more specific about what you’re calling ‘connection’? The term ‘polyphony’ suggests that things not only come together but that they sound together. So, what connection makes the music?

**Rex**
Each of the papers is doing something unique, but we also bring out the commonality of our work, the connection between, as I say, the author writing about somewhere from somewhere else.

**Renate**
I think this has been happening for a long time, we just didn’t look at it. And are we now looking at it any differently? I want to say that this discussion we’re having captures our process of discovery. To me, this process is an emphasis on *how* we do things, which to me is really important and often neglected. So changing our focus on the interconnections makes the difference, for example, with regard to the transnational histories Rex just mentioned. And my question certainly is this: should we reflect on the *how* in a more focused way? The question of doing otherwise, especially with regard to colonial paradigms of doing knowledge production and doing research, is what we are engaged in here right now as well. So, my sense would be to bring the idea of play into this format and ask how we can find different ways of doing this kind of debate, offering space for different people to find different answers, depending on the contexts they’re looking at, to have different kinds of conversations and see what evolves – then you get the specificity rather than an overarching view. It is like kind of looking at it from a metaphorical grassroots point of view, bringing out all sorts of different points of view quite literally as our papers are all grounded in different geographies and historical contexts, bridging moments between them. I would say it is not just about the relationships which have always been there but about developing them, asking what this means for doing art history. Writing these histories is the first stage of creating archives; then the question is what you do with the archive as it builds.

**Khadija**
I think this is an important aspect – to resist the archival impulse to archive those connections or those relationships in a certain kind of art history writing that this paragraph in Astrid’s paper points to:

The Doctrine of Discovery was used to separate so-called civilized and primitive peoples, and to give the former custodianship over land inhabited by the latter.^9^ We can argue that the logic of preservation is an extension of this doctrine, as it is used by museums and archives to justify the extraction of images and objects from other lands and transfer them for care in Western institutions. The underlying assumption, still present in restitution discussions today, is that looted objects and images are safer in modern, scientific settler-colonial institutions than in the lived environments of their cultural origins.

That the logic of preservation is linked to early modern Catholic missionary doctrines resonated with research I’m doing at present into the politics of blocking restitutions through conservation restrictions. In the example I’m writing about (*The Contested Crown: Repatriation Politics between Mexico and Europe*, 2022), it is claimed that the technologies with which the museum in Vienna can better preserve an Aztec feather headress are more secure and valuable than what would change if it returned to its place of origin. So-called hard science plays a powerful role in such arguments, as does the moralising of Christianity.^10^ Elizabeth Povinelli and I talked about this in our contribution to this special issue as also being a mode of reformation that Western culture repeatedly enacts to assimilate but not cede power to those outside its borders.

**Figure:**
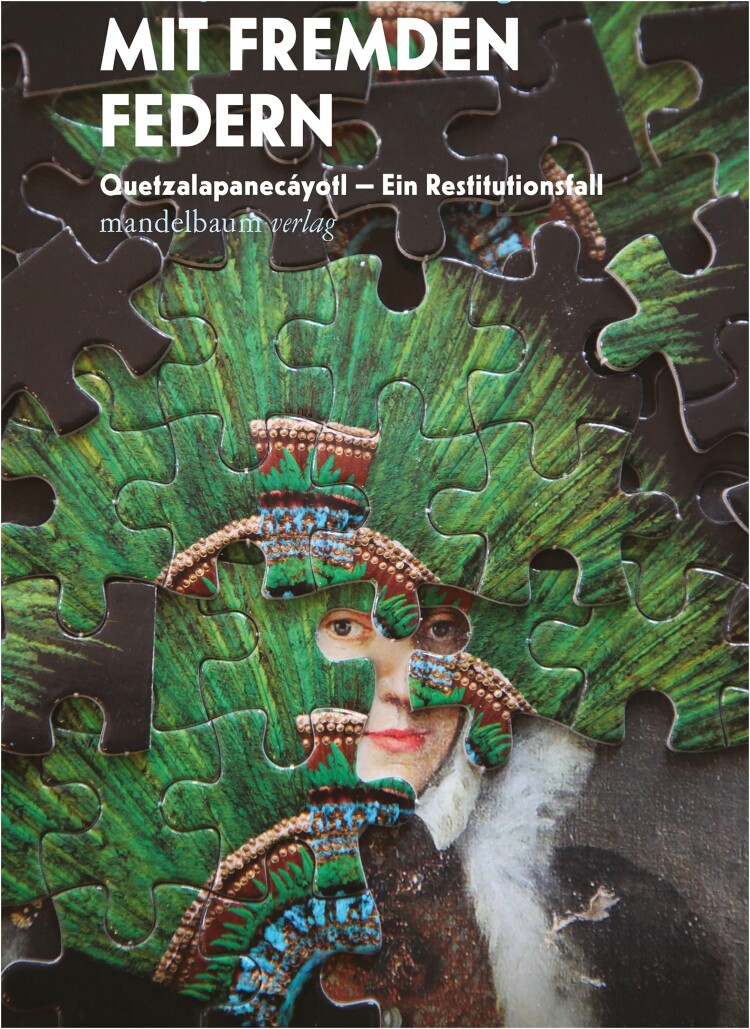
Khadija von Zinnenburg Carroll, cover of *Mit fremden Federn*, German edition of The Contested Crown, 2022

**Azadeh**
I’m thinking about why we have chosen polyphony to describe our special issue; what is its definition for us within writing art history? I’m wondering whether the term stands for both consonant and dissonant voices; if yes, can polyphonic art history cover contradictions and negotiations in a broad sense? or do we need to be more specific and perhaps reflect on where polyphony is supposed to be manifested? We are polyphonic in our positions, utterances, relations, presumptions, arguments, and in our methodologies. Also, we used the word ‘harmony’ in relation to polyphony and to describe the commonalities between our papers. As Renate rightfully questioned that, I’m thinking about polyphonic art history as something that also includes or refers to disharmonious relations or conversations between different narratives, artworks, artists.

**Renate**
Maybe just to add to that the idea of resonance, if we stay with the musical paradigm, having different tonalities, or does everything we say have a different tone and add up to something that is bigger? There can be dissonance of course, as well, depending on what kind of musical pieces develop; it doesn’t have to fit together because to me this is part of the old paradigm, but things can be more fluid and move in different ways. Here sound is a good analogy: sound travels, it swells and fades.

**Deniz**
I agree with Renate, and I think that it’s important not to try to fix it, because then we’re doing exactly what we’re trying to undo, and with the archives we rather see that it’s a living thing. Reading Renate’s comments on my paper I was also struck by the kaleidoscope and maybe how that’s something visual but how that relates to polyphony and is perhaps a translation of a similar concept?

**Khadija**
Just to continue the metaphor, a kaleidoscope is made up of many fragments and when you move it, it always rearranges its relationships and then creates a new image, so it is also like the moving images of us on this screen. This screenshot of our Zoom call shows a visual polyphony in action.

**Figure:**
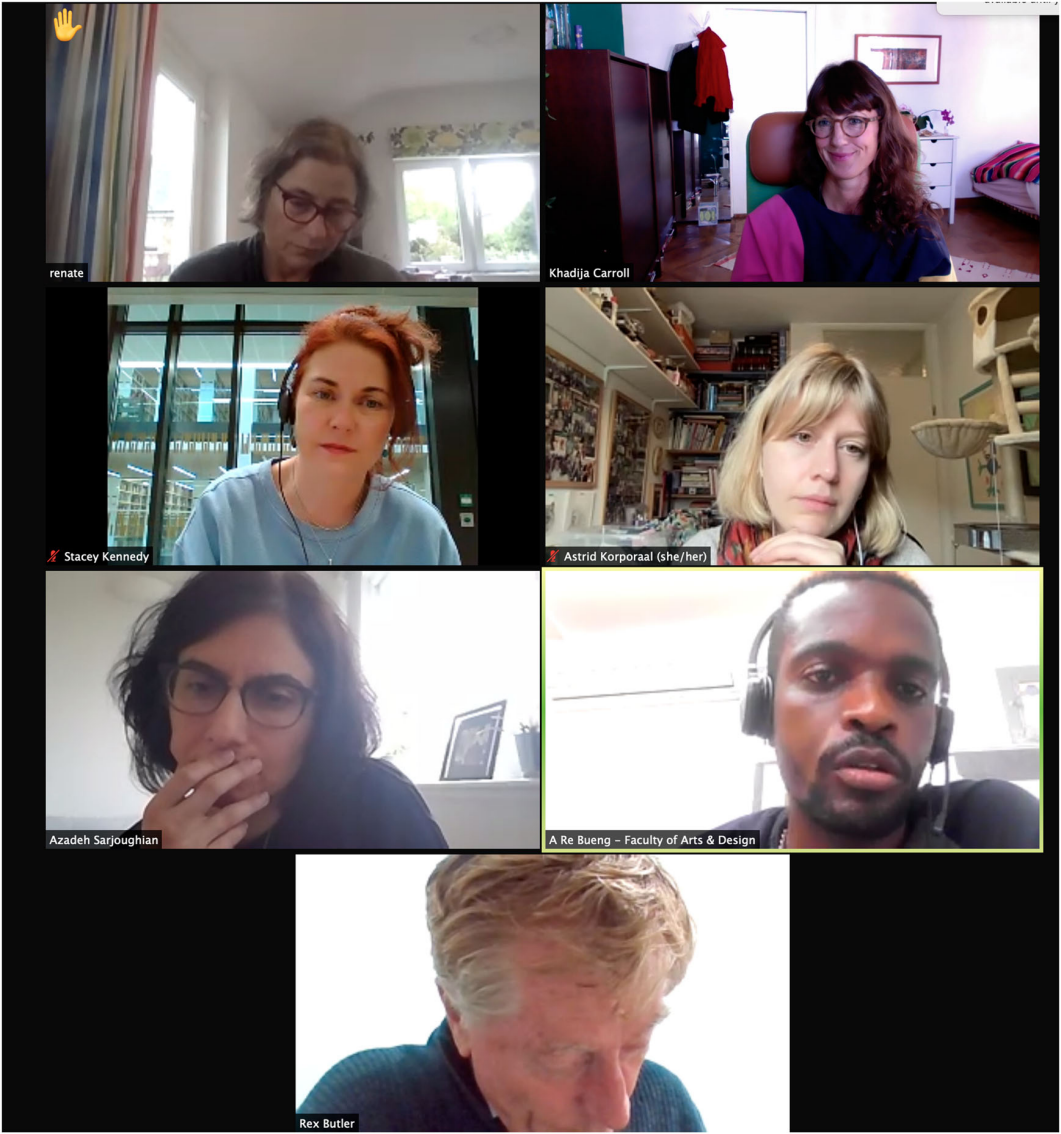
Khadija von Zinnenburg Carroll, screenshot of roundtable discussion

**Khadija**
We might even describe these modalities in our attempt to describe the *how* of what we’re *doing*. There are various artists that have written about that from the perspective of artistic research.

**Astrid**
I wanted to respond to Deniz, who quotes in her paper: ‘standing on shifting ground makes it clear that every view is a view from somewhere and every act of speaking is an act of speaking from somewhere’.^11^ And I thought this idea of standing on shifting ground was relevant, because we’re talking about connections between places but we’re also talking about undoing or questioning national borders as definitions of place. Perhaps by multiplying them or moving differently with them.

**Rex**
That is a good quote because I think one of the points we would be making is that, unlike those globalist world histories, there’s no totality that these essays add up to. There is just a series of perspectives and there’s no underlying world that they are the perspective on to. I think you would pose in the editorial introduction the paradox that these are a set of voices that do not add up to a totality or a choir singing from the same score.

**Astrid**
Maybe part of the ground is the idea that one can return and shift in time as well as in place. Because I think there’s also the subject of healing, trauma and care in several of the texts. This relates to the problem of when you say it’s all just different perspectives, then it can seem relativist. But in our pieces there’s also the sense of responsibility and the need to respond, which is also about polyphony.

**Barbara**
I am interested in the aspect of listening. Polyphonic art history is not about creating or discovering polyphony, because this would be very modern thinking again.^12^ The point is to assume that a polyphony has always been there but has not been sufficiently perceived, at the level of both methods and the object of research. I really liked the concept of peripheral listening that Astrid brought up in her text, where she states: ‘Peripheral visiting involves stepping over an imaginative spatial threshold and having to reorient and retune the senses in response to surroundings which are not one’s own.’

**Khadija**
Like a call and reply, which exists in music. Someone sings and someone then follows on – a modality exists that’s not called polyphony.^13^

**Astrid**
Looking up the etymology of ‘polyphony’, it comes from the Greek *polyphōnia*, meaning ‘having many sounds or voices’, so it is not even necessarily musical. And maybe this is the connection to build on. It brings in a non-Western perspective and the notion of opacity. We’re listening but we don’t understand everything; the sounds and words are not totally transparent.

**Azadeh**
Yes, it is important to acknowledge the right to opacity and avoid the expectation of complete coherence within these transcultural relations. For me, polyphony is also a reference to Hermans’s notion of ‘dialogical self’ as Herman was inspired by Bakhtin’s idea of the polyphonic novel, and there he talks about the self as a heterogeneous society.^14^ So, we can trace polyphony within the landscape of the mind too, within our internal dialogues, as within our minds we may experience a plurality of unmerged voices, similar to what happens in our social relationships. I tried to explore the metaphor of polyphony in my paper when I discuss Neshat’s *Land of Dreams* as the artist entering her subjects’ dreams in the process of recording and collecting them. The work is basically a depiction of intersubjective exchanges between the artist and her subjects based on their sharing of traumatic experiences caused by various historical events. But it all seemed to be happening in the unconscious realm. I think this dreamy aesthetic helped to keep the opacity of the subjects’ stories and emotions.

**Renate**
It is fabulous to think about the archiving of dreams as well, because it adds to the notion of the archive in really interesting ways, which also touches on what Monkman does to a certain extent. By bringing in different levels of vision, imagination, dream, myth, whatever you want to call it, you bring in levels that have often been written out of history or shoved into separate disciplines of the humanities to a certain extent. I think the issue of the dominant ideology that informs the archive is raised quite often, but there are also efforts to archive forgotten histories, so the notion of the archive is versatile, multifaceted and complex. It functions in different ways depending on the specificity of the context. If it’s an archive of work by anti-apartheid photographers, for example in Marie’s research, it is important to create and is counter-colonial. Other archives are thoroughly colonial.^15^

**Astrid**
To respond to this idea of multiple selves or multiple voices within the self, I think that relates to the relationship to place and a communal sense of self. This is also a refusal of a more Western colonial way of thinking that we could even speak as an individual self. Acknowledging that it’s not even fully transparent to ourselves how we are connected to other voices and other places. I think that’s also something that we’re trying to work through in our thinking and writing.

## Collective Endeavours and Persistent Individuals

**Astrid**
What I have noticed is that art history is often still working with the modern idea of the individual artist, but if you think about contemporary art, there is much more interest in collectives: artists as social activists or as social enablers.

**Stacey**
Yes, interestingly all of the recent (2021) Turner Prize (UK) nominees have been art collectives. But thinking about the ethnographic art collection here at Birmingham University as an archive, I would come at this from the opposite perspective. So often the historical presentation of African art in Europe has suffered a homogenising trope of primitivism which constructs African art production as a collective endeavour. This leads to the unclear categorisation of art objects that confuses groups of people and places. For example, art being labelled as a creation of ‘the Dogon’ or ‘the Yoruba’, which you still see in museums today. It’s not about saying we can’t trace named artists within the Danford Collection, there are amazing Yoruba sculptures by Bámigbóyè and Lamidi Fakeye, but their individual biography can be overlooked because the Danford Collection is typical of the ethnographic collection, which relies on the primitivist construct to interpret the art it contains. I want to spotlight one artist because that’s the way I’m trying to approach troubling, or decolonising, the collection. This painting by Clara Etso Ugbodaga-Ngu, a really great example of modernism in Nigeria, has been placed in the Danford Collection alongside items like shoes and bags and furniture, located among what I would call utilitarian objects. Suddenly you’ve got this important painting lost among these other items, suffering from a framing which affects how we read it, how we value it in art history.

This echoes Linda Nochlin, who outlines the construct of the ‘great artist’ in Western art history as an individually talented Italian male.^16^ An artist worthy of extensive biography. I try to be careful with the idea of anonymity, almost pushing against it, to argue that research into named female Nigerian artists is important to reveal the marginalised ‘great artists’ of art history. Speaking with contemporary artists in Nigeria, they say they want to be recognised as talented individuals in ways they were denied in the past.

**Figure:**
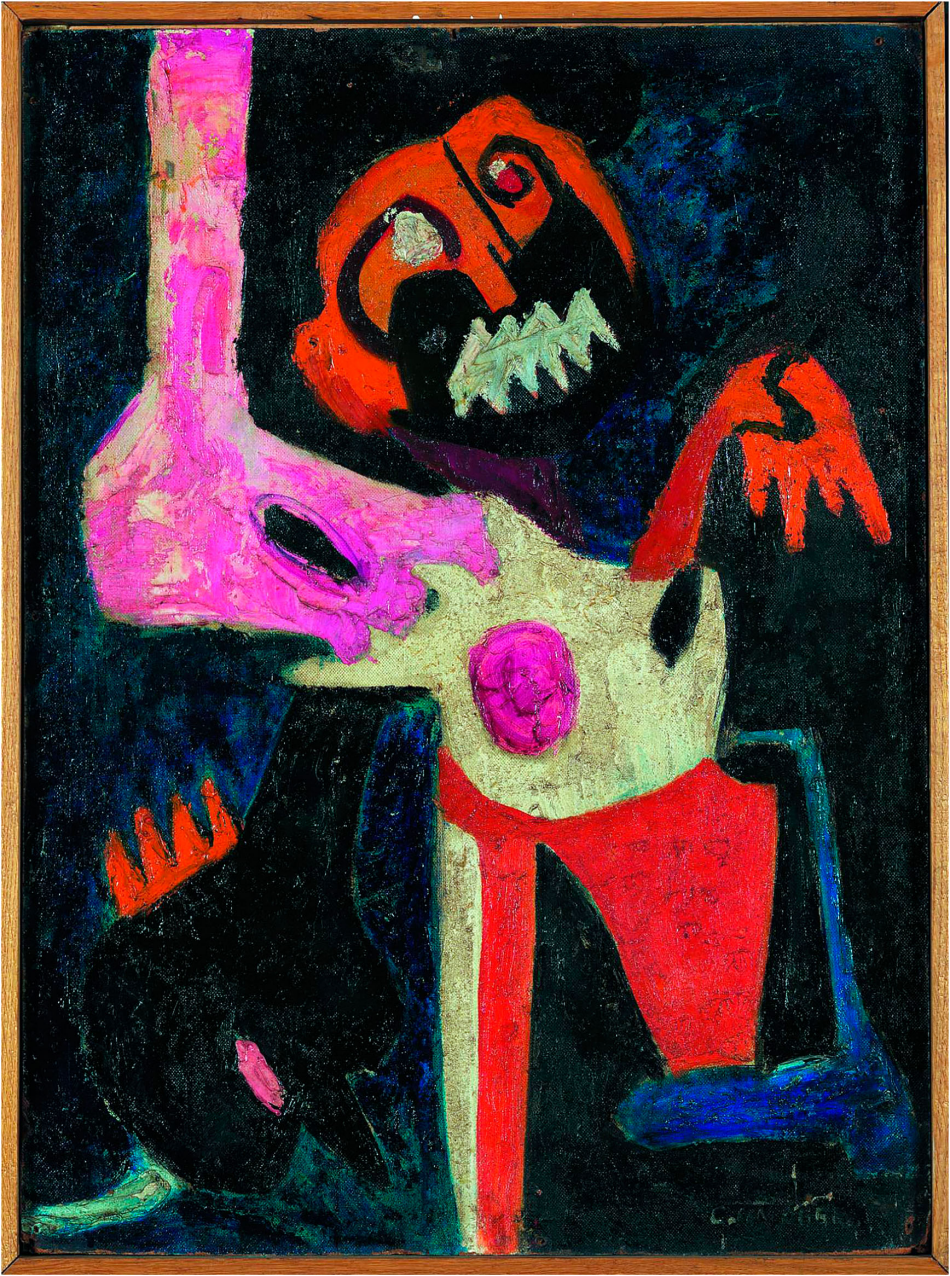
Colette Omogbai, *Agony*, 1963, oil on masonite, IwalewahausDEVA, Universität Bayreuth, © Colette Omogbai

**Khadija**
So the problem is that it’s a reaction, right? A reaction to being erased. And sometimes it’s impossible to reclaim names that weren’t recorded, names that are absent in the archives. Rather than excessively trying to counteract that erasure, one approach might be to speculate on the collective. For me, it’s quite freeing to consider that in some contexts the pressure to produce individually is lifted and artistic practice becomes something pedagogical and collective.^17^

**Astrid**
I was interested in how Stacey’s article showed the influence of the artist as part of a particular scene, showing a sign of her genius in a way that adds to the idea of the genius as an individual.

**Rex**
I think collectives are far too conscious, but artists do always work with others in an art ‘scene’. They can’t work by themselves, they necessarily work with other people around them. But in art history we individualise artists, and even though we talk about art movements, when we hang artworks on museum walls they are invariably understood as the work of one artist without trying to recreate the scene out of which they come. The fact is that virtually no one does their work by themselves. Several of our papers are about particular artistic scenes from around the world, and it’s the scene that brings people together. It’s not necessarily an explicit idea, just a collection of people that come together for some reason or another. It’s not a matter of a manifesto or collective movement, all those words tend to be just retrospective.

**Renate**
I would want to include the imagination of a scene here, or its reimagination, as happens in Monkman’s work. One must be careful not to project a particular understanding of what it means to be an artist on to history, because this runs the danger of being Eurocentric and ends up exercising a kind of violence. And, thinking about the Indian context, I wanted to throw in that this notion of an individualised Western artist that Stacey talks of, quoting Nochlin, was hugely attractive to Indian practitioners in the late nineteenth and early twentieth centuries as a goal and status to reach. The notion of the individual genius, which was not part of the Indian context of visual culture, was very attractive. Ravi Varma was the first Indian artist to achieve this new status of fine artist in the late nineteenth century. One must be careful about terms and how we use them, and be wary of making assumptions.

**Pfunzo**
We must locate the names of individual Black artists, especially women. This is our art-historical burden of sorts. But the point Rex is making here is that in the process of acknowledging the artist who Stacey writes about, Ugbodaga-Ngu, as a phenomenal artist, we must also write the history of the other artists who enabled her to shine. Speculatively, Ugbodaga-Ngu was perhaps the product of an invisible hand of a greater community of African women artists from the era. One could say that she is the tip of the iceberg. So yes, let us put her name up in lights, but also recognise that she’s part of a scene or group of other women artists who will never make it into the limelight. We should use her name to speculate on this community of African women artists who were certainly working at the time.

**Stacey**
Yes, the idea of the exceptional *woman* could be limiting; perhaps we should think about about exceptional *women*, recognising their individuality within the collective. Regarding Ugbodaga-Ngu’s influence and the art scene she was a part of, we know that in 1958 she was a teacher at NCAST, so she likely influenced the artists who formed the Zaria Art Society. Art historian Chika Okeke-Agulu centres the Zaria movement and their concept of national synthesis as the crucible for postcolonial modernism in Nigeria, but Ugbodaga-Ngu is overlooked in that history. Other female artists were working across Nigeria around the time of independence, for example Constance Afiong Ekong and Colette Omogbai. They worked in collaboration with the British Council, the colonial administration and cultural hubs such as the Mbari Club, to form strategic alliances and gain a kind of strategic agency.

**Renate**
The fact that she taught the Zaria Rebels is important. It also raises the question of matriarchal structures in Nigerian culture, but I wouldn’t know the specificity. I wonder whether this perhaps made it possible for her to be so influential, with nobody challenging her authority because she was teaching. I think this could well be underscored; it is worth noting even if we don’t know more about it.

**Stacey**
Art historian Itohan Osayimwese says we’re looking for great women artists in the wrong places and we’re not going to find great Nigerian women artists if we keep on looking in the same places. This means that we need to look at women as teachers, because we know that women often haven’t got the means to become full-time studio artists and must take on second jobs. If they find themselves teaching then that often becomes the primary lens with which they’re viewed, and they’re seen as secondary artists because they were educators. I am arguing the reverse and saying, no, hang on a minute, if you’re educating other artists who’ve gone on to become the greatest artists, you are also in that category of great! This is a polyphony we must untangle and expand if we want to escalate the voices and talents of women artists from anywhere, not just Nigeria.

**Renate**
And to me that creates another sense of the polyphony we’re talking about, the artists’ scene. Approaching the issue from the point of view of the Western notion of the individualised artist, one would look at the group and forget about her, but really she’s fundamentally part of this even though she’s not literally part of it. So again, it’s kind of rethinking how we frame things, what narratives we bring to bear.

## Footnotes

1 Markus Messling and Jonas Tinius, eds, *Minor Universality/Universalité mineur: Beyond Universalism/Partager l’universel*, De Gruyter, Berlin, 2023, https://doi.org/10.1515/9783110798494

2 **Deniz** The notion of polyphony is important here, because in our heads there are all these voices that we’ve digested by reading or talking to people, whether things have been voiced to us directly or been filtered. I can definitely connect to polyphony. Whereas the transnational seems like its been there for a long time, but of course it is still very important, as you mention it in the introduction. In any case, it will be great to turn our focus to the multiplicity.

3 Khadija von Zinnenburg Carroll, ‘Re-membering the Figure: The Ekphrasis of J J Winckelmann’, *Word & Image Journal of Verbal and Visual Inquiry*, vol 21, no 3, 2005, pp 261–269

4 Ian Burn, ‘Is Art History Any Use to Artists?’, *Art-Network*, no 15, Autumn, 1985, pp 9–14

5 Museum of Archaeology and Anthropology, ‘The Power of Paper: 50 Years of Printmaking from Australia, Canada and South Africa’, 14 February – 6 December 2015, https://maa.cam.ac.uk/power-paper-50-years-printmaking-australia-canada-and-south-africa, accessed 14 November 2023

6 Édouard Glissant, *Poetics of Relation*, University of Michigan Press, Ann Arbor, Michigan, 1997, p 20

7 Michael Herzfeld, ‘The Politics of Gesture: Ethnographic Reflections on the Embodiment of Globalization’, provisional paper, accessed 27 December 2023, http://ceas.iscte.pt/ethnografeast/papers/m_herzfeld.pdf

8 **Azadeh** Thinking about our bodies’ reactions to moments of resonance is fascinating! What you say here also recalls what Hartmut Rosa says about resonance as an unpredictable experience. Still, we know that to experience it, we need to situate ourselves in a particular position in relation to our surroundings. I think what makes experiencing Deniz’s artwork and writing a resonant experience is that it evokes a feeling of caring about rituals and root identities, rather than preserving or conserving them.

9 The Doctrine of Discovery was based on Pope Alexander VI’s 1493 decree that any land not inhabited by Christians was available to be discovered, claimed and exploited by Christian rulers. ‘Inter Caetera’ Demarcation Bull, 4 May 1493, Gilder Lehrman Collection.

10 Khadija von Zinnenburg Carroll, ‘Risk Reassessment and Safe Transportation for the Penacho’, Repatriates, https://repatriates.org/mexico/risk-reassessment-and-safe-transportation-for-the-penacho/, accessed 29 November 2023

11 Lila Abu-Lughod, ‘Writing against Culture’, in Richard G Fox, ed, *Recapturing Anthropology: Working in the Present*, School of American Research Press, Santa Fe, New Mexico, 1991, p 141

12 **Khadija** (**ZT** *mother*) But I always thought of the Modern as Father rather than Mother?!

13 **Renate** It’s more experimental jazz or something!

14 Hubert J M Hermans, ‘The Dialogical Self as a Society of Mind: Introduction’, *Theory & Psychology*, vol 12, no 2, 2002, pp 147–160

15 **Khadija** I think the archive might be like a song. There’s the archive of dreams from us – that’s the kind of short song that comes up – and polyphony is more the method or modality, the *how* that we discuss, and the archive is the *what*.

16 Linda Nochlin, ‘Why Are There No Great Women Artists?’, in Vivian Gornick and Barbara Moran, eds, *Woman in Sexist Society: Studies in Power and Powerlessness*, Basic Books, New York, 1971, pp 480–510

17 **Astrid** But we shouldn’t overlook individual names in the process, correct? **Khadija** No, absolutely not. It’s not about erasing individual contributions.

